# Comparison of trace image colors for kids-book with two active distractions in reducing pain and fear of children during the venipuncture procedure

**DOI:** 10.1007/s00431-023-05271-y

**Published:** 2023-10-14

**Authors:** Sherzad Khudeida Suleman, Nizer Bakir Yahya, Stefan Nilsson, Karin Enskär

**Affiliations:** 1 Department of Women’s and Children’s Health, Uppsala University, Uppsala, Sweden; 2 Psychiatric and Pediatric Nursing Unit, College of Nursing, University of Duhok, Duhok, Kurdistan Region Iraq; 3 Pediatric Medicine Unit, College of Medicine, Duhok University, Duhok, Kurdistan Region Iraq; 4 Institute of Health and Caring Sciences, University of Gothenburg, Gothenburg, Sweden

**Keywords:** Child, Fear, Pain, Distraction, Non-pharmacology

## Abstract

This study investigated the effectiveness of trace image and coloring for kids-book (TICK-B), cough trick, and balloon inflation techniques in reducing pain and fear in children during venipuncture. The current study is a prospective, controlled, and randomized trial (RCT). School-aged children who required venipuncture were involved in the study. Pediatric patients were randomly assigned to four groups: the TICK-B group, the cough trick group, the inflation of balloons, and the control groups. Before and after the procedure, the children and their parents were interviewed. Wong-Baker (FACES) Pain Rating Scale was applied to measure the severity of pain. Children’s Fear Scale was applied to measure children’s fear. This study involved the 160 children (mean age, 8.39–2.18 years). The severity of pain and fear levels among the children during and after the procedure were significantly different (*p* = 0.001). Pain and fear were significantly decreased in children in the intervention groups compared with those in the control group (*p* < 0.05). In the TICK-B group, participants reported significantly less pain and fear during the venipuncture procedure than in the cough trick, balloon inflation, and control groups (*P* = 0.001, *p* = 0.001, *p* = 0.001) and after the procedure (*p* = 0.001, *p* = 0.002, *p* = 0.002). There was a similar significance found in the level of fear during the procedure (*p* = 0.001, *p* = 0.002, *p* = 0.006), and after the procedure (*p* = 0.001, *p* = 0.008, *p* = 0.015).

*Conclusion*: TICK-B was the most effective method for decreasing the pain and fear of children associated with venipuncture procedures. Furthermore, the distraction technique of coughing and inflating balloons also proved efficacious in decreasing the pain and fear of children during venipuncture.

*Trial registration*: The study has been registered with ClinicalTrials.org under the number NCT04983303. It was retrospectively registered on July 26, 2021.

**Table:** 

**What is Known:** *• Venipuncture, one of the most painful and uncomfortable procedures for children, caused great fear and discomfort during the procedure.*	
**What is New:** *• The TICK-B technique, music listening, and cartoon watching techniques are effective, simple, and safe ways to reduce children’s fear and pain. These interventions provide a good way for children and their parents to collaborate during painful medical procedures.* *• No studies have compared the impact of TICK-B during venipuncture.*

**What is Known:**

*• Venipuncture, one of the most painful and uncomfortable procedures for children, caused great fear and discomfort during the procedure.*

**What is New:**

*• The TICK-B technique, music listening, and cartoon watching techniques are effective, simple, and safe ways to reduce children’s fear and pain. These interventions provide a good way for children and their parents to collaborate during painful medical procedures.*

*• No studies have compared the impact of TICK-B during venipuncture.*

## Introduction

Venipuncture is routine in pediatric patients’ settings for blood collection and IV catheterizations [1], which can cause pain and fear [2, 3]. According to Humphrey et al. [4], A majority of children aged 7 to 12 who had their venipunctures reported feeling distressed during the process. Anxiety and fear are commonly associated with needle-related procedures. Anxiety seems to be an important factor in predicting the experience of pain. Psychological factors have a variety of effects on pain perception [5]. According to the literature, stress, anxiety, and fear may not only increase pain, but may also cause it [6, 7]. Needle-related procedures are particularly stressful for young patients. Frequently, children express concerns prior to undergoing the procedure that anticipatory fear may increase pain and lead to distress on an emotional level [8]. Anxiety and stress are frequently associated with needle-related procedures and can cause catastrophizing and avoidance behavior (needle phobia) [9]. While this is true, only 10% of painful procedures are performed with pain management as part of the procedure [10]. Children who do not receive adequate pain and fear management during needle procedures may have anxiety at follow-ups and may experience needle phobias throughout their lifetimes [11, 12]. Therefore, effective pain relief methods must be used when children undergo needle procedures.

The management of pain includes both pharmacologic and non-pharmacologic strategies that have been applied to decrease the pain and fear of children during painful procedures [13, 14]. The most common pharmacological approach for decreasing pain from medical procedures is topical anesthetic creams [14]. Topical anesthetic creams induce local anesthesia but require a 45 to 60 min waiting period [14]. A variety of non-pharmacological techniques are effective in dulling the sensation of procedural pain when applied appropriately [14].

Non-pharmacological strategies include many different activities, such as an inflating balloon and a cough trick [15]. Non-pharmacological methods have a number of advantages, including reduced pain and fear, as reported by parents, children, and observers [16]. Therefore, it is very important for children to be provided with effective methods of pain relief during needle procedures [17].

Distraction diverts children’s attention away from harmful stimuli in a simple and effective manner. However, a variety of technologies and techniques are associated with distractions [18]. Distraction is a non-pharmacological technique that is extremely effective in decreasing children’s pain and fear during painful procedures. Distraction can be active or passive [17, 19].

Active distraction is an approach in which one or more of the child’s senses and skills are stimulated during painful procedures so that the child can engage in certain assignments. Active distraction is often employed in clinical practice in the form of controlled breathing, interactive toys, guided imagery, virtual reality, and electronic games [18]. While in passive distractions, children do not participate in activities during procedures; healthcare professionals actively distract them. In pediatric patients, the most commonly used passive distraction techniques are music and television [18]. The effectiveness and benefits of distraction have been demonstrated by children, their parents, or observer nurse reports on decreasing pain and fear in children [20].

Art therapy is an effective method of distraction. Using this technique, children can cope with stress before, during, and after painful procedures [21]. There is a technique called trace image and coloring for kids-books (TICK-B) that has been developed for use in psychological intervention through the use of art. The goal of this technique is to decrease the pain and fear of children during venipuncture procedures. There are many benefits associated with this technique, and it is one of the most cost-effective, attractive, easy, and simple to apply during medical procedures for children [22, 23].

Cough trick (CT) is a method of pain relief during peripheral venipuncture (VP) and various injection procedures that involves coughing in response to commands simultaneously with skin puncture [24]. Coughing causes an increase in intrathoracic pressure, which in turn stimulates the autonomic nervous system to increase blood pressure and heart rate, increase subarachnoid pressure, and increase baroreceptor activity [25]. The pain-inhibiting pathways in the segmental brain are activated by increasing the subarachnoid pressure; therefore, a combination of increased blood pressure and activating baroreceptors seems to have a substantial effect on diminishing pain perception [26].

In addition to actively distracting attention from unpleasant situations, balloon inflation and coughing can also have physiological effects [25, 27]. In balloon inflation, venous return is reduced due to an increase in intrathoracic pressure [27]. An increase in pressure may stimulate baroreceptor activation in conjunction with pulmonary vessel contraction, and it has been suggested that activating cardiopulmonary and sinoaortic baroreceptor reflex arcs can relieve pain [27].

This study investigated the impacts of TICK-B, inflation balloon, and cough trick on school-age children during venipuncture procedures. The research hypothesized that children who applied TICK-B, balloon inflation, or the cough trick during their venipuncture procedures would experience less pain and fear than those who did not.

In our study, TICK-B, balloon inflation, and cough trick were hypothesized to result in lower levels of pain and fear during venipuncture than the control group, and TICK-B showed greater effect than balloon inflation and cough trick.

## Methods

### Study design

The study was done at the Pediatric Teaching Hospital in Kurdistan Region/Iraq between November 1, 2022, and January 20, 2023. The study was a prospective, randomized, parallel-group trial of pediatric patients requiring venipuncture procedures conducted in a double-blind form.

### Setting and sample

The present study included school-aged children aged 6–12 years who were hospitalized at Heevi Pediatric Teaching Hospital. The study participants included 160 randomly chosen children who met the eligibility criteria. For eligibility, pediatric patients must be 6–12 years old and require venipuncture. Children with neurodevelopmental delays, speech difficulties, impairment of hearing or vision, or who had taken analgesics in the previous 6 h were excluded.

### Interventions

#### TICK-B

TICK-B consists of pictures that require coloring. The participants were given TICK-B before venipuncture began, and they were instructed to draw or color a picture during the procedure as they preferred [22, 23]. Examples of this intervention are shown in Fig. 2.

#### Balloon inflation

In this group, after they had undergone a latex allergy test, the children selected the color of their balloons. Initially, the balloons were inflated once to assess their suitability for inflation. The participants took balloons before the procedure began and were instructed to inflate them during the procedure [15, 17].

#### Cough trick

Each child was taught how to cough during venipuncture by the first author. In preparation for the procedure, children were instructed to take breaths deeply and cough actively. When a child learns how to cough properly, his or her procedure begins. The pediatric patients were instructed to inhale prior to the needle being inserted and to cough throughout the procedure. In this group, the children were informed that during venipuncture procedures they would cough [15, 25].

### Control group

In this group, the children were permitted to remain near their families. A routine blood collection procedure was conducted.

### Instruments

The Child Information Form, the Wong-Baker Scale (FACE Scale), the Children’s Fear Scale (CFS), TICK-B, balloon inflation, and the cough trick were all used to collect data.

#### Child and family information form

In a pre-designed questionnaire, the general characteristics of pediatric patients were recorded, including age, gender, length of hospitalization, and number of injections.

#### Wong-Baker FACES pain scale (WB-FACES)

The scale of WB-FACES was applied to evaluate the severity of pain in children based on their self-reports. The scale ranged from 0 to 10. It consisted of six different illustrated faces, demonstrating different emotions, from 0, very happy, no pain to 10, which hurts way worse (crying) [28].

#### Children’s Fear Scale (CFS)

The scale of CFS was applied to measure the level of fear in children during venipuncture procedures based on their facial expressions. It ranges from 0 to 4. It includes five facial expressions. Each facial expression is given a score. In the case of the face picture without reaction (0 points), the child is not afraid, while the face picture of the child who is scared (4 points) indicates that the child is extremely afraid [29].

### Sample size

In order to estimate the sample size for the study, a power analysis was conducted. Based on a previous study by Suleman et al. [22], the intervention group had a standard deviation of 1.5, and the control group had a standard deviation of 2.0. According to a power of 80 and a Type I error size of 0.05, each group required 40 participants. To prevent missing data due to technical difficulties, approximately 10% of the sample size was increased.

**Fig. 1:**
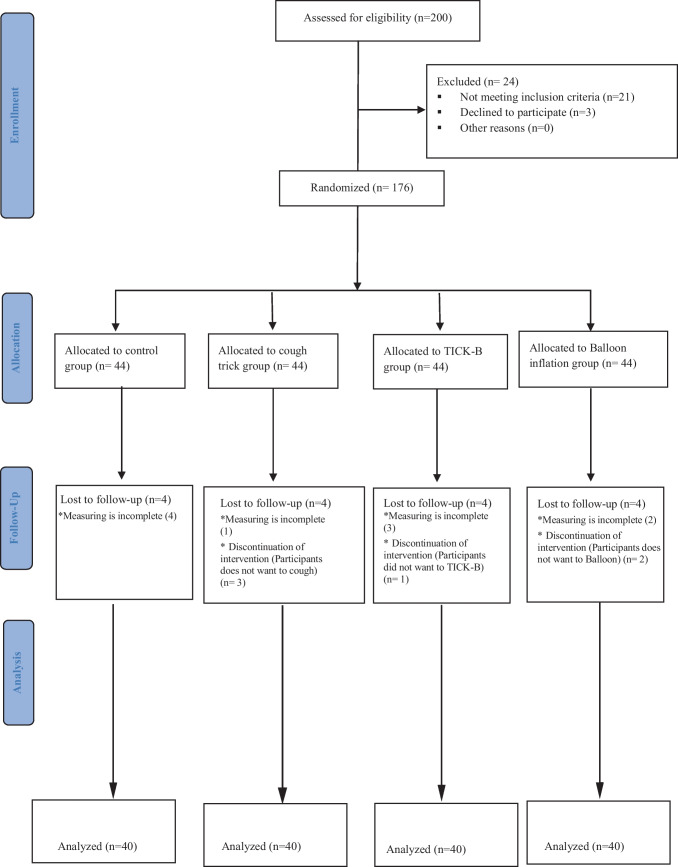
Flow chart showing the recruitment of patients for the study

The children were randomly divided into four groups: 40 were assigned to the TICK-B group, 40 to the balloon inflation group, 40 to the cough trick group, and 40 to the control group (Fig. 1). The envelope technique was used for simple randomization. The patients received opaque sealed envelopes containing information assigned to a group. The children and their parents were interviewed face-to-face after the procedure to collect all outcomes.

### Randomization

A predesigned form was used to record the names of patients requiring venipuncture. This study used simple random sampling to ensure blindness and randomization. Using a computer-generated sequence, eligible participants were randomly allocated to four groups. We sequentially numbered opaque and sealed envelopes. In a sealed envelope, information about patients is contained that assigns them to one of four groups. Each child was asked to pick out one of these envelopes. Of the 176 patients who agreed to participate, four groups were randomized. Each group included 44 patients. The children were randomly divided into four groups: 44 were assigned to the TICK-B group, 44 to the balloon infusion group, 44 to the cough trick group, and 44 to the control group. Four children from the control group did not have their samples tested due to being in a hurry. From the cough trick, one child had unmeasured outcomes while three others refused to cough. In the TICK-B group, three children had unmeasured outcomes, and one refused to color. In the balloon inflation group, two of the participants had unmeasured outcomes and the other two refused to inflate the balloon.

The randomization envelopes for each intervention were assigned by the ward nurse, who was blinded to the study. Families and children were informed of the group to which they had been assigned. The opaque sealed envelopes blinded nurses, parents, and children to groupings. The allocation was concealed by randomly assigning one child to each study group from each room in the emergency department. In addition, we coordinated with the emergency ward’s head nurse to prevent anyone from entering the room and interrupting. The observer nurse, the children, and their parents were not informed of the main purpose of the study, and the interventions were performed on the patients during venipuncture procedures. This observer is a senior pediatric nurse in the emergency department who was trained in pain and fear assessment by the second author but was not involved in the study design or statistics analysis and had no knowledge of all procedures performed in the study.

The venipuncture procedure was performed by a clinical performance nurse with extensive pediatric nursing experience. Nurses were masked from the study’s main objective of the interventions, all assessments, and comparisons of outcomes between participants. To allow comparison between groups, children were asked to rate their pain and fear levels after venipuncture without being informed of the grouping. Additionally, the clinical nurse and parents of the children were instructed not to reveal the nature of the intervention to their children. Therefore, the children were unaware of the aims of the intervention (Fig. 1).

### Procedure of study

In this study, a clinical performance nurse with more than 8 years of experience working in pediatric patient settings and needle procedures conducted the venipuncture procedure. No conflicts of interest were reported by the nurses. Pediatricians make clinical decisions regarding patients’ venipuncture. The researcher read parents and children a piece of brief information about the pain and fear tools before randomization, and both acknowledged understanding the instructions. To evaluate the level of pain and fear experienced by pediatric patients before and after the venipuncture procedure, a self-report form was used: the 0–10 WB-FACES scale for pain and the 0–4 CFS scale for fear. For all children, the second nurse performed the venipuncture procedure. Following the venipuncture procedure, the children were asked to assess their fear levels and pain severity. There were 160 children randomly assigned to four groups of 40 using opaque, sealed envelopes. After group assignments, the parents and children went to the venipuncture room.

**Fig. 2:**
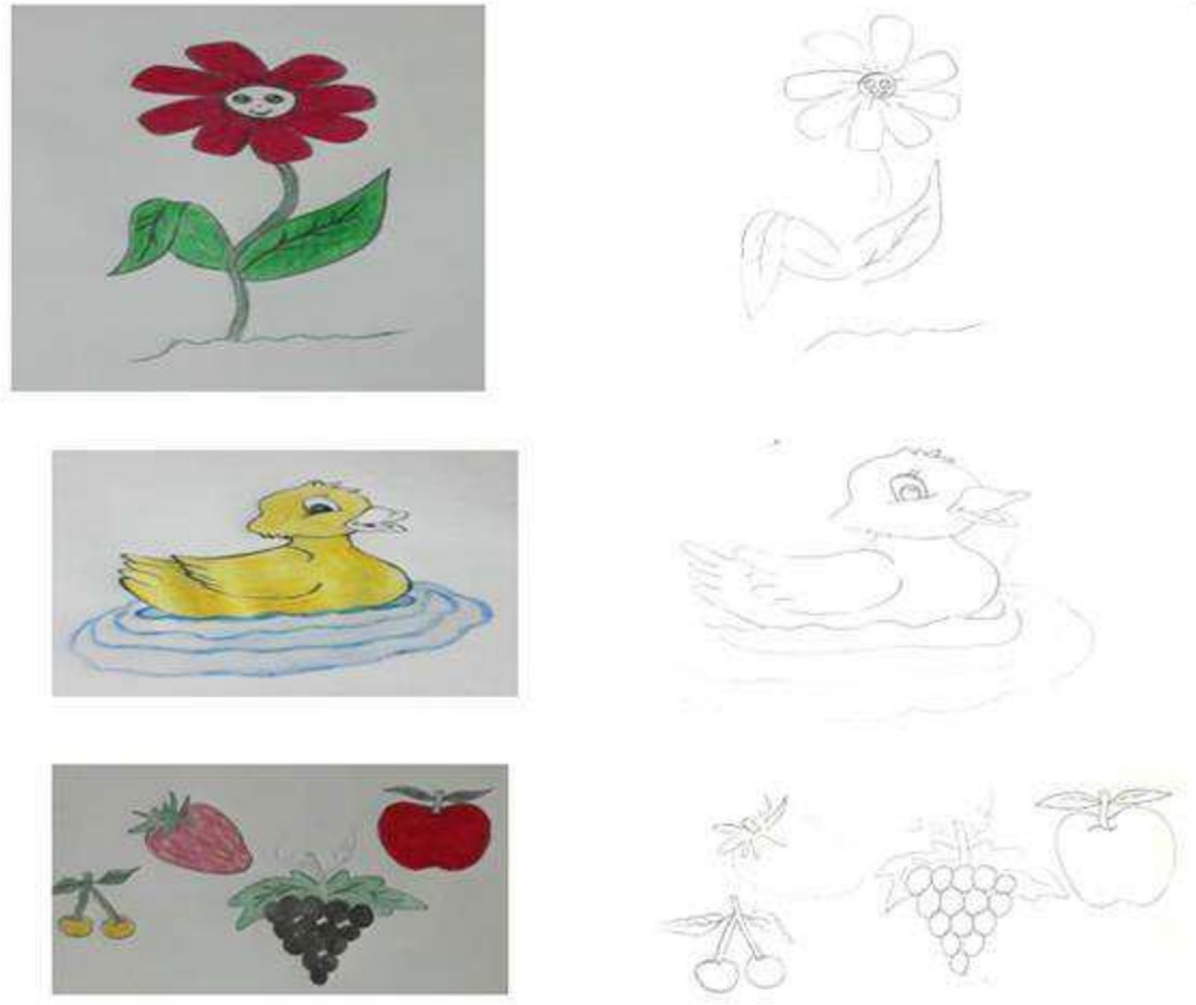
Example of photos used for the intervention of “Tracking Images and Coloring Children’s Books” (TICK-B)

Venipuncture sessions were performed between 8:00 and 12:00 a.m. with a 5-ml injector and a 22-G needle. Parents remained with their children during venipuncture. Children in the intervention groups, prior to needle insertion, were instructed to cough, inflate the balloon, or color a picture during venipuncture procedures (Fig. 2). They were instructed to focus on their intervention and to maintain to do so until the venipuncture procedure was completed. The level of pain experienced by the children after the procedure was measured in the same manner as their fear levels. The performance nurse used the same needle size for all children. By self-reporting, face-to-face interviews were conducted.

### Statistical analyses

The statistical analyses were conducted using the Statistical Package for the Social Sciences version 28.0 (SPSS Inc., Chicago, IL, USA). Means, standard deviations, frequencies, and percentages were used to summarize the descriptive data. The Shapiro-Wilk *W* test was used to determine the normal distribution of the Wong-Baker FACES and Child Fear Scale scores. Chi-square tests and *t* tests were used to analyze all parametric data and baseline characteristics. For parametric data, such as the level of pain and fear of the children, a one-way analysis of variance was used. The significance level of the study was set at *p* 0.05, and a Bonferroni test was performed as a post hoc analysis.

### Ethical considerations

The study was part of a Ph.D. The paper was approved on June 1, 2021, by the Duhok General Health Directorate unit of the Scientific Research Directorate (registration number 01062021-5-1). Publication rights were granted to this study (reference number: 01062021-5-1). It also received administrative approval from Heevi Hospital. According to the Declaration of Helsinki, the parents of all children participating in the study were required to provide written informed consent prior to their children entering the study. No harmful interventions were performed in this study.

## Results

This study included 160 (50.63 (81) girls, 49.37 (79) boys). The age range of the children was 6 to 12 years old (mean = 8.39) and SD = 2.183. The pediatric patients were assigned into four groups: control (*n* = 40), cough trick (*n* = 40), balloon inflation (*n* = 40), and TICK-B (*n* = 40). The baseline characteristics of the children are presented in Table 1: age, gender, attempts, hospitalization length, previous pain, and pre-procedural fear levels were similar among the groups. There was no statistically significant difference between the study groups in terms of self-reported pain and fear levels (*p* = 0.945 and *p* = 0.971, respectively).

**Table 1:** A comparison of baseline characteristics and preprocedural fear scores among the study groups

**Child’s characteristics**	**Groups**				***p*** **value (two-sided)**
	**Control group (*n* = 40)**	**Cough trick group (*n* = 40)**	**Balloon inflation group (*n* = 40)**	**TICK-B group (*n* = 40)**	
**No. (%)**					
Gender					**0.831**
Male	20 (50.0)	18 (45.0)	22 (55.0)	19 (47.5)	
Female	20 (50.0)	22 (55.0)	18 (45.0)	21 (52.5)	
Mean (SD)					
Age	8.55 (2.06)	7.65 (2.15)	8.43 (2.39)	8.93(1.97)	**0.226**
Attempts	1.15 (0.36)	1.20 (0.40)	1.18 (0.38)	1.10 (0.30)	**0.073**
Hospitalization days	3.35 (1.00)	3.53 (0.81)	3.35 (1.00)	3.60 (0.95)	**0.412**
Previous pain	7.05 (1.69)	6.48 (1.82)	6.48 (1.82)	6.83 (1.72)	**0.945**
Pre-venipuncture fear	2.48 (0.87)	2.55 (0.93)	2.55 (0.93)	2.55 (0.93)	**0.971**

The severity of pain experienced during and after venipuncture is shown in Table 2. According to self-reports, there was a significant difference in child pain intensity between the groups during and after venipuncture (*p* = 0.001) and after the procedure (*p* = 0.001). In comparison to the control group, the TICK-B group showed a significant decrease in pain severity, cough trick, and balloon inflation during and after the procedure (*p* = 0.001, *p* = 0.001, *p* = 0.001, respectively) and after the procedure (*p* = 0.001, *p* = 0.002, *p* = 0.002). Similarly, the coughing trick group and the balloon inflation group significantly decreased in experienced pain compared to the control group during and after the procedure (*p* = 0.010, *p* = 0.000) and after the procedure (*p* = 0.000, *p* = 0.000). There were no differences between the levels of pain with balloon inflation and the cough trick during the venipuncture procedure (*p* = 0.140) or after the procedure (*p* = 1.000).

**Table 2:** The severity of pain among the groups

Child’s characteristics	Groups				*p* value	*p*					
	**Control group (*n* = 40)**	**CT group (*n* = 40)**	**BI group (*n* = 40)**	**TICK-B group (*n* = 40)**		TICK -B vs. control	CT vs. control	BI vs. control	TICK-B vs. CT group	TICK -B vs. BI group	CT vs. BI group
	***M* = SD**					*p* value					
Pain during procedures	7.15 = 1.68	5.85 = 2.22	4.93 = 1.80	2.88 = 1.50	0.001	0.000	0.010	0.000	0.001	0.001	0.14
Pain after procedures	7.08 = 1.67	4.10 = 1.61	4.10 = 1.61	2.78 = 1.44	0.001	0.000	0.000	0.000	0.002	0.002	1.00

Wong-Baker Pain Rating Scale

*BI* balloon inflation, *CT* cough trick, *TICK-B* trace image and coloring for kids-book

Table 3 presents the levels of procedural fear experienced during and after venipuncture. Based on the self-report, there was a significant difference between the groups in the level of fear during venipuncture among children (*p* = 0.001) and similarly after the procedure (*p* = 0.001). In the TICK-B group, the fear was reduced significantly during and after the procedure than in the control, cough trick, and balloon inflation groups (*p* = 0.000, *p* = 0.002, *p* = 0.006, respectively) and after the procedure (*p* = 0.000, *p* = 0.008, *p* = 0.015). Similarly, the coughing trick group and the balloon inflation group significantly reduced the fear of the control group during and after the procedure (*p* = 0.006, *p* = 0.002) and after the procedure (*p* = 0.009, *p* = 0.005). There was no significant difference between the levels of fear with the balloon inflation and the cough trick during the venipuncture procedure (*p* = 1.000) and after the procedure (*p* = 1.000).

**Table 3:** Comparison of fear scores between the groups

Child’s characteristics	Groups				*p* value	*p*					
	**Control group (*n* = 40)**	**CT group (*n* = 40)**	**BI group (*n* = 40)**	**TICK-B group (*n* = 40)**		Tick-B vs. control	CT vs. control	BI vs. control	TICK-B vs. CT group	Tick-B vs. BI group	CT vs. BI group
	***M* = SD**					*p* value					
Fear during procedures	2.66 = 0.78	2.00 = 0.94	1.94 = 0.97	1.27 = 0.80	0.001	0.000	0.006	0.002	0.002	0.006	1.00
Fear after procedures	2.53 = 0.87	1.86 = 0.98	1.82 = 0.12	1.18 = 0.71	0.001	0.000	0.009	0.005	0.008	0.015	1.00

Child Fear Scale (CFS) fear rating scale

*BI* balloon inflation, *CT* cough trick, *TICK-B* trace image and coloring for kids-book

## Discussion

Pain experienced during procedures, such as venipuncture and cannulation, can lead to fear, which can continue to negatively affect the well-being of pediatric patients throughout their lifetimes [30]. The American Society for Pain Management Nursing suggests that pain management needs to be provided in the most optimal manner, both before and during painful procedures [31]. In the last few years, non-pharmacological and distraction methods based on cognitive and psychological behavior, particularly, have been widely employed during venipuncture procedures [32].

In the literature, there is ample evidence that distraction is effective in reducing the level of pain and fear experienced by children during painful procedures [32]. In several studies, distraction methods have been evaluated by parents and health professionals as a means of decreasing pain and fear associated with painful medical procedures, and these techniques have been found to be effective [17, 33–35].

The use of distraction techniques can be effective, easy to access, inexpensive and safe. In this study, balloon inflation, cough trick, and TICK-B were evaluated as distraction techniques. Numerous studies ([15, 17]) have demonstrated cough tricks in decreasing the pain and fear caused by needle procedures. This study found, as in previous studies, that the cough trick significantly decreased pain and fear levels compared with the control group during venipuncture. In different studies in the literature, it has been demonstrated that inflating balloons during painful medical procedures helps reduce pain and fear [15, 17, 33]. The results of a study by Sahiner and Bal [33], which found that school-age children in a balloon inflation group experienced less pain and fear during intravenous access procedures than those in a control group, provided an effective approach to painful medical procedures. According to our study, the balloon inflation group showed reduced pain and fear levels when compared with the control group.

In two recent studies, Suleman demonstrated that TICK-B is effective in alleviating children’s fear and pain during venipuncture procedures among school-aged children, establishing that it is a safe and effective distraction technique [22, 23]. In our studies, we found that the TICK-B method had the same effect as those two studies in terms of reducing children’s pain and fear levels among school-aged children. According to the study, this intervention reduced children’s fear and pain during venipuncture when compared to the control group, cough trick, and balloon inflation. A study done by Mutlu and Balcı [15] found that the inflation of balloons did not reduce fear and pain in children during venipuncture procedures statistically significantly more than coughing tricks did. In addition, the current study found that balloon inflation and coughing tricks were equally effective in reducing the pain and fear experienced during venipuncture by children.

One of the benefits of TICK-B is that distraction can help the child focus his or her attention away from unwanted stimuli during needle procedures, such as needle insertion. There is no doubt that coloring pictures as a part of an art-based therapy activity, such as TICK-B, provided enjoyable entertainment for children at this age and proved to be helpful in distracting them during venipuncture procedures [22, 23]. This method of distraction was based on the simplicity of how nurses can apply it and children can enjoy it. Furthermore, it is less time-consuming and more cost-effective. Thus, TICK-B might be useful as a distraction method for alleviating pain and fear during painful medical procedures. The evidence for the effectiveness of distraction with TICK-B on pain and fear control is limited in the literature [22, 23]. Recently, two studies examined the use of TICK-B to distract children during venipuncture using two different scales: the WB-FACES and the visual analog scale [22, 23]. In their studies, TICK-B was effective in controlling pain and anxiety during painful procedures.

According to research published in the literature, pain and fear ratings have not been affected by some demographic characteristics such as gender, age, attempt, and hospitalization period. The results of our study confirm these findings [22, 23]. In the present study, all the characteristics were similar between the studied groups.

In addition, TICK-B demonstrated greater effectiveness than cough trick, balloon inflation, and no distraction in controlling self-reported procedural pain and fear. In general, it has been accepted that pediatric patients who have experienced a painful procedure in the past may experience fear in the future as well. For this reason, it is a fundamental aspect of pediatric clinical practice to reduce the emotional consequences of venipuncture procedures with better pain management to minimize psychological effects. To avoid the negative consequences of painful procedures in the future, successful pain management must be given the utmost attention.

### Strengths and limitations

In addition to the use of randomized controlled trials (RCTs) and triple blinding in our study, we also have a sufficient sample size, which is an important strength of the study. In our study, 160 children participated, making it the most extensive experiment ever conducted on various types of distractions for decreasing venipuncture procedure pain and fear in children. This study was designed to blind nurses, assessors, statisticians, and children from the main purpose of the study order to ensure fairness. Despite this, there are still some limitations to the generalization of our results. First, the children participating in this study were all hospitalized in a single hospital. The second limitation of the present study was that it only included school-age children. Therefore, the results may not be representative of those in other hospitals.

## Conclusion

The TICK-B method has been found to be the most effective approach to decreasing pain and fear during and after the venipuncture procedure in school-aged children compared to balloon inflation and coughing tricks. Nurses should be aware of the pain associated with venipuncture procedures and the fear they can evoke in patients. In order to decrease the pain and fear experienced by children during venipuncture, distraction methods such as TICK-B, balloon inflation, and cough tricks should be utilized. This study contributes to the body of knowledge about nonpharmacological pain management strategies; however, the results should be replicated in other settings.

## Data Availability

On reasonable request, the corresponding author will be provided with the data supporting the conclusions of this article.
